# High-speed camera recordings uncover previously unidentified elements of zebrafish mating behaviors integral to successful fertilization

**DOI:** 10.1038/s41598-021-99638-6

**Published:** 2021-10-12

**Authors:** Buntaro Zempo, Natsuko Tanaka, Eriko Daikoku, Fumihito Ono

**Affiliations:** 1 Department of Physiology, Osaka Medical and Pharmaceutical University, Takatsuki, 569-8686 Japan; 2 Division of Integrative Physiology, Department of Physiology, Jichi Medical University, Shimotsuke, 329-0498 Japan

**Keywords:** Physiology, Zoology

## Abstract

The mating behavior of teleost fish consists of a sequence of stereotyped actions. By observing mating of zebrafish under high-speed video, we analyzed and characterized a behavioral cascade leading to successful fertilization. When paired, a male zebrafish engages the female by oscillating his body in high frequency (*quivering*). In response, the female pauses swimming and bends her body (*freezing*). Subsequently, the male contorts his trunk to enfold the female’s trunk. This behavior is known as *wrap around*. Here, we found that *wrap around* behavior consists of two previously unidentified components. After both sexes contort their trunks, the male adjusts until his trunk compresses the female’s dorsal fin (*hooking*). After* hooking*, the male trunk slides away from the female’s dorsal fin, simultaneously sliding his pectoral fin across the female’s gravid belly, stimulating egg release (*squeezing/spawning*). Orchestrated coordination of *spawning* presumably increases fertilization success. Surgical removal of the female dorsal fin inhibited *hooking* and the transition to *squeezing*. In a neuromuscular mutant where males lack *quivering*, female *freezing* and subsequent courtship behaviors were absent. We thus identified traits of zebrafish mating behavior and clarified their roles in successful mating.

## Introduction

Zebrafish is a freshwater teleost whose natural habitat is shallow water in South Asia^1,2^. As a widely used model organism in biomedical research^3^, the mechanism of zebrafish reproduction has been studied for both ethological and practical reasons, such as maximizing egg collection for experiments^4^. In natural environments, zebrafish spawn in shallow water with aquatic vegetation^1,2^. In the laboratory, mating tanks are designed to emulate the natural environment and elicit *spawning* behaviors^5^. Upon successful mating, a single female zebrafish produces up to 200–300 eggs in a single *spawning* session^6^. Depending on the quality of eggs and sperm, typically 58–78% of released eggs become fertilized^7^.

The description of mating behavior in zebrafish is variable among the literature^2,8–10^, and recent technologies now offer the opportunity to better understand the intricacies of this behavioral cascade. One commonality, however, is how activation of (room) daylight initiates a male’s pursuit toward a female into the shallow area of the mating tank. Heretofore, mating behavior has generally been characterized by the following steps: *undulate, chase, escort, encircle, quivering, pin, wrap around,* and *spawning*^5,8,11^. In context, male and female fish swim back and forth within their habitat for tens of minutes (*undulate*). At some point, the male begins to follow the female closely (*chase*). The male appears to guide the female to* spawning* sites by repeatedly swimming between the female and the shallow areas of the mating tank (*escort*). The pair swim together in circles (*encircle*), and the male oscillates his body close to the female (*quivering*). Occasionally, the pair appear to court while in contact with the wall (*pin*). The male enfolds the female with his trunk (*wrap around*), and both sexes release their gametes (*spawning)*. Kang et al. called the *wrap around* step *grasping*, highlighting the role of the male pectoral fin positioning, in conjunction with trunk alignment between the pair^12^.

**Figure 1:**
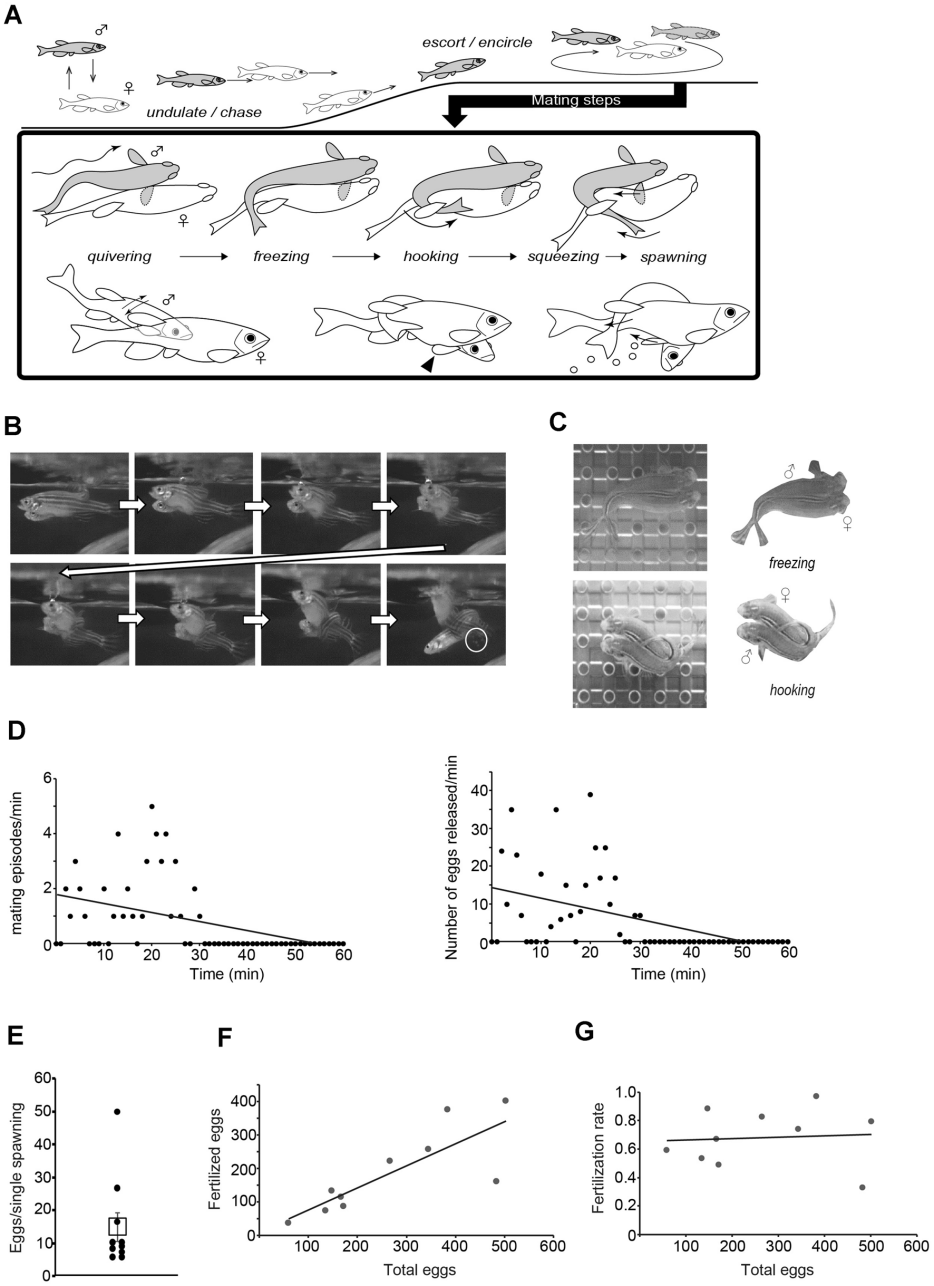
Analyses of mating process in intact WT zebrafish pairs. (**A**) A schematic illustration showing the entire process of mating. The male fish is shown in grey. The expanded panel represents processes from *quivering* to *spawning*. In *hooking*, one pectoral fin of the male is positioned under the female belly (arrow head). Arrows in illustrations represent directions of fish movements. (**B**) Photographs from a video showing the process from *freezing* to *spawning*. The male fish is behind the female in the initial frame. Released eggs are encircled with a white line in the last frame. Arrows indicate temporal sequence. (**C**) Photographs of *freezing* (upper) and *hooking* behavior (lower). In *freezing*, the female bends her body in S-shape in response to *quivering*. In *hooking*, the male holds the female using the female dorsal fin as an anchor for its contorted trunk. (**D**) Representative time course displaying the number of mating events (left) and eggs released (right) in 1 min during a 60 min pairing session. (**E**) Total egg numbers were divided by the numbers of *spawning* in a single pairing session (n = 10 pairs). Dots represent values of individual pairs. A box represents the accumulated data. (**F**, **G**) The number of fertilized eggs and fertilization rate were plotted against the total number of eggs. The number of fertilized eggs strongly correlated with the total egg number (r = 0.81, *p* = 0.0047). Fertilization rate was independent from the total egg number. Regression lines are shown in (**D**), (**F**) and (**G**).

The details of each mating behavior step, and their specific contributions toward successful fertilization, have not been clarified to the extent possible. Here, we explored zebrafish mating behavior in fine detail and classified our new observations within the existing step (Fig. 1A); we focus on the significance of individual steps using surgical manipulations and mutants.

## Materials and methods

### Fish lines and maintenance

The RIKEN-WT strain and the nicotinic acetylcholine receptor ε subunit knockout (εKO) line (ZFIN ID:ZDB-ALT-201215-9) were used in the present study. Zebrafish were maintained in self-circulating systems at Osaka Medical and Pharmaceutical University (OMPU) and Jichi Medical University (JMU). This study was carried out in compliance with the ARRIVE guidelines. All methods were carried out in accordance with relevant guidelines and regulations. All experimental protocols were approved by Institutional Animal Care and Use Committees at OMPU and JMU.

### Observation of mating and counting of eggs

Zebrafish were kept under long day conditions (8 AM–22 PM light). On the night preceding the observation, a pair consisting of one male and one female fish (3–4 months old; n = 10 unique pairs were used) were placed in a mating tank (Tecniplast, Buguggiate, Italy), separated by a divider. Standard length and body weight were 30.3 ± 0.9 mm, 630 ± 73 mg for males, 28.0 ± 1.6 mm, 406 ± 78 mg for females. The following morning, the divider was removed at 8 AM and mating behaviors were documented over the following hour. Mating behaviors were recorded for 60 min by both a home video camera HDR-CX420 (Sony, Tokyo, Japan) and a web camera DC-NCR300U (Hanwha Q CELLS Japan, Tokyo, Japan), positioned to capture both a horizontal and vertical view of the mating tank. *Quivering, hooking,* and *spawning* during the initial 30 min were manually identified and quantified off line, based on the slow-motion replay of the recorded videos. At 9 AM, we collected all eggs, then counted total eggs and fertilized eggs at 5–7 h post fertilization (hpf). Any eggs developed past the epiboly stage^13^ met criteria as fertilized eggs. In experiments using εKOs, we extended the mating behavioral observation to 3 h, to account for the possibility that the mutant pairs exhibited delayed* spawning* (requiring > 1 h).

### Dorsal fin removal

After anesthetizing adult female fish (3–4 months old; 10 fish) in 0.04% tricaine (Tokyo Chemical Industry, Tokyo, Japan), dorsal fins of female zebrafish were removed (> 80% of the original length) with surgical scissors. After 2 days of recovery in a tank, the surgically altered female and an intact male were paired for mating. Mating behaviors were recorded for 1 h or 3 h (εKO).

### High-speed video analysis of mating behavior

High-speed image capturing of wild type (WT) pairs, WT male and dorsal fin-removed female pairs, and WT and εKO zebrafish pairs were performed with a Photron camera (Photron, Tokyo, Japan) at 1000 frames/s. For the analyses on *quivering*, captured images were saved in JPEG format and processed with ImageJ. For each male performing *quivering* behavior, we measured the series of head angles as described in Zempo et al.^14^ as a proxy for plotting trajectory over time. We calculated and statistically analyzed amplitudes between the minimum and the maximal peaks of head angles during the initial 110 ms of *quivering*.

### Statistical analysis

**Figure 2:**
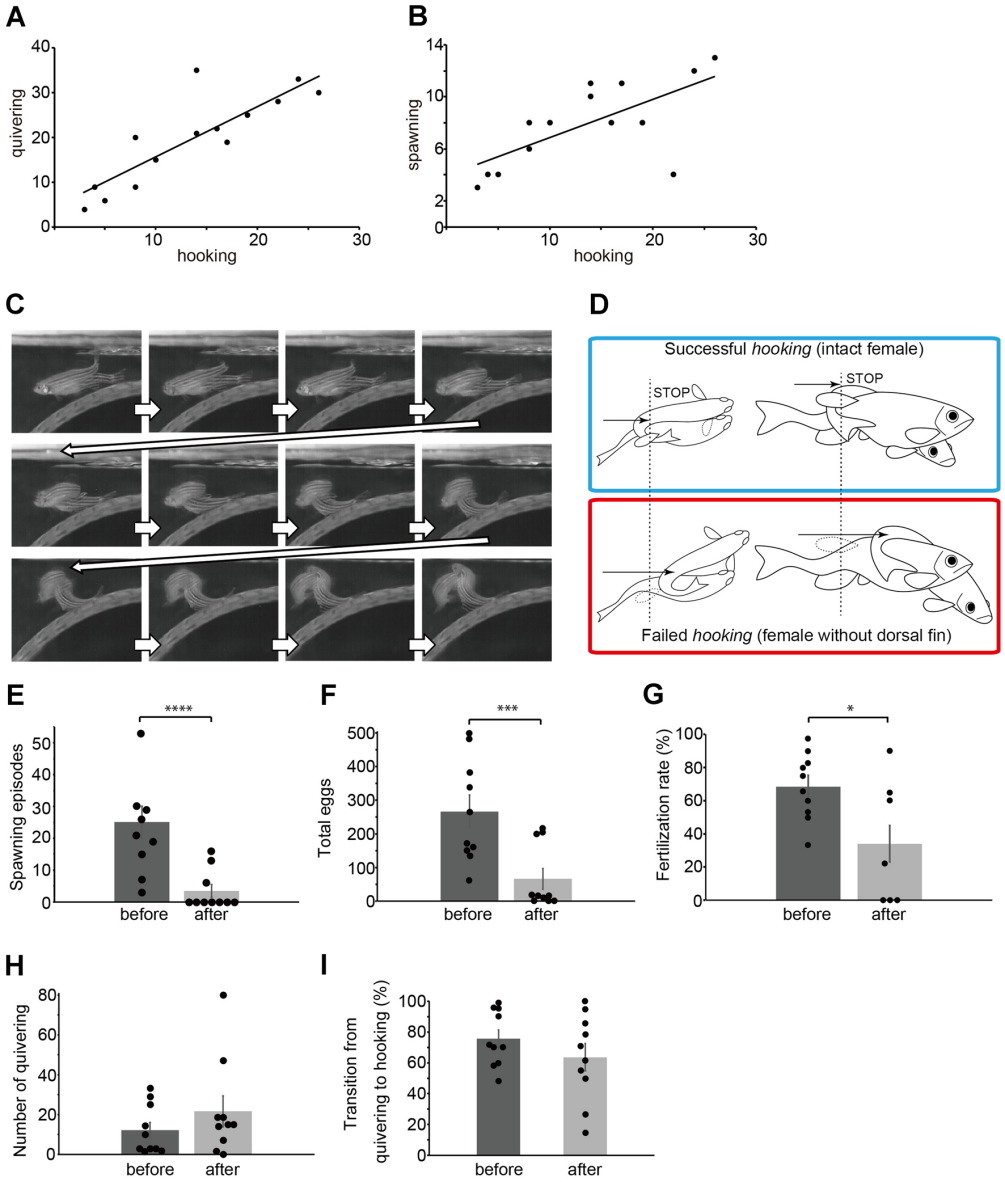
Functional significance of *hooking* behavior. (**A**) The numbers of *quivering* plotted against those of *hooking*. (r = 0.85, *p* = 0.00010). (**B**) The numbers of *spawning* plotted against those of *hooking* (r = 0.68, *p* = 0.0069). (**C**) Photographs showing mating behavior of an intact male and a female with its dorsal fin removed. The female is behind the male in the initial frame. The trunk of the male slips rostrally without switching to *squeezing*. (**D**) Illustrations showing typical *hooking* behavior of a WT intact pair (upper blue box), and the WT pair with the female fin removal (lower red box). (**E**) Identified episodes of *spawning* before and after the fin removal (*p* = 0.00088, t-score = 3.98, n = 10). (**F**) Total number of released eggs before and after the fin removal (*p* = 0.0029, t-score = 3.44). (**G**) Fertilization rate before and after the amputation. Fertilization rate was significantly decreased after dorsal fin removal (*p* = 0.027, t-score = 2.45). (**H**) Episodes of *quivering* before and after the fin removal (*p* = 0.29, t-score =  − 1.08). (**I**) The percentage of pairs proceeding from *quivering* to *hooking* (Note all *hooking* bouts finished incompletely after the fin removal, *p* = 0.27, t-score = 1.15). Regression lines are shown in (**A**) and (**B**).

**Figure 3:**
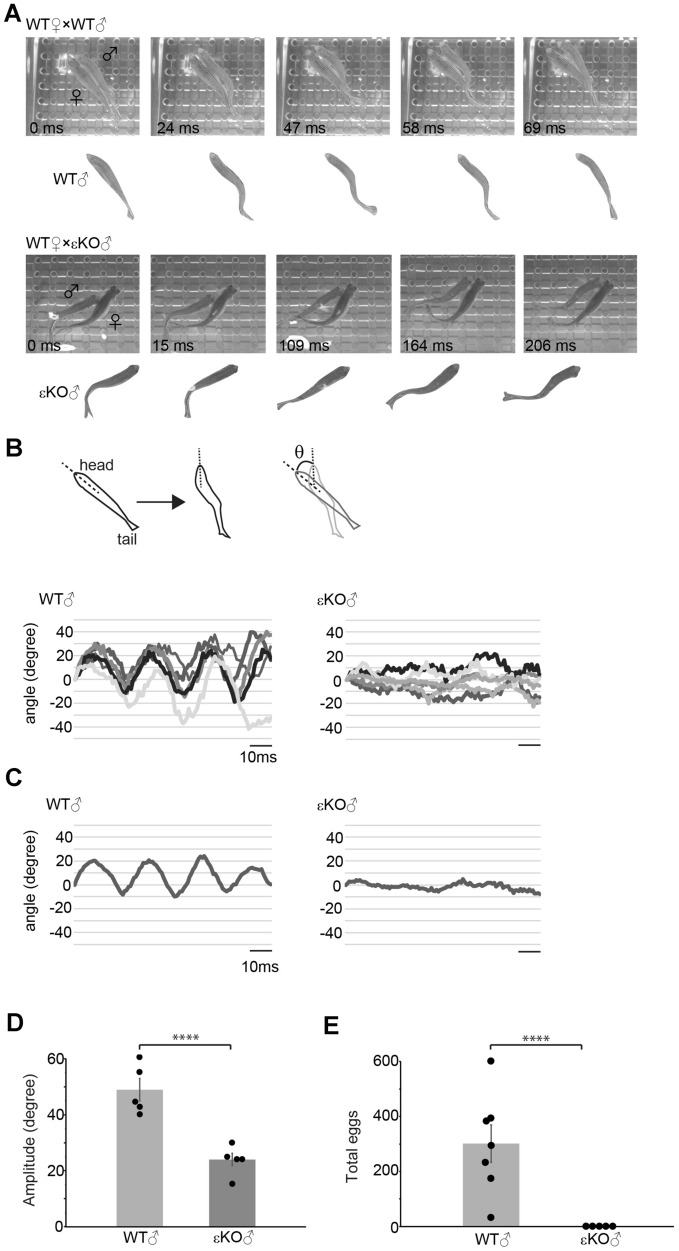
Analyses of *quivering* using εKO males (**A**) Photographs showing the *quivering* behavior of the WT male x WT female pair (upper) and the εKO male x WT female pair (lower). A cropped image of the male provided below each photograph for clarity. (**B**) Kinematics for representative traces of head turns in the WT male and the εKO male. Schematic illustrations in the upper panel shows measurement of head angles. Lines represent outlines of a fish, and the head angle θ is obtained from broken lines. 0° corresponds to a straight body. In the lower panels, each trace represents a different male (n=5 each). Head angles are shown in degrees, with 0 indicating a straight body, and positive and negative values indicating body bends in opposite directions. Scale bars: 10 ms. (**C**) Traces shown in (**B**) were averaged. (**D**, **E**) The amplitude of head turns (*p* = 0.00060) (**D**) and the total number of released egg (**E**) (*p* = 0.00086) compared between the WT male x WT female and the εKO male x WT female pairs.

In Figs. 2E–I and 3D, E, unpaired t test (two-tailed) was performed for statistical analysis. For correlative analyses, Pearson product-moment correlation coefficient was calculated and the statistical significance was analyzed by Student's t-distribution.

## Results

We used a high-speed camera (1000 frames/sec) to capture behavior immediately preceding *spawning* and examined coordination of mating sequences leading to successful fertilization (Fig. 1A, B, Movies 1 and 2). Based on the framework of Sessa et al.^5^ and Kang et al.^12^, we classified steps of mating as shown in Fig. 1A, with modifications as described below.

Following, and perhaps in response to, *quivering* by the male, the female stalls swimming and twists her trunk slightly; we call this step *freezing* (Fig. 1C and Supplemental Fig. 1). We propose separating the period formally known as “*wrap around*” into two separate steps based on the forward-then-inverse direction of male trunk movement relative to the female trunk. We call the first step, in recharacterizing *wrap around*, *hooking. Hooking* involves the male contorting his trunk, wrapping it around the female’s trunk, and sliding it rostrally (Fig. 1C). As reported in Kang et al.^12^, one pectoral fin of the male was also positioned under the female belly. After contacting the female dorsal fin, the male appears to apply mechanical pressure to the trunk of the female with his trunk and pectoral fin (Movies 1 and 2). Notably, the female moves forward during this process, allowing the male trunk and pectoral fin to shift caudally along the female body. We call this step *squeezing*, the timing of which partially overlaps with that of *spawning*. Males presumably release sperm during or immediately after *squeezing* which facilitates one completed cycle of mating.

To investigate the importance of these newly found behavior steps, we first analyzed mating episodes, clutch size, and fertilization success in mating between WT zebrafish. Time course analysis showed that mating episodes (one episode defined by one set of sequential *quivering, hooking* and *spawning* behaviors) concentrated in the initial 30 min following the initial *quivering* behavior; mating episodes were sparce after 40 min (Fig. 1D). During *spawning*, the number of eggs released per unit time also showed a similar pattern (Fig. 1D). Given this time course, we quantified behavioral events for 30 min in subsequent analyses, while eggs were collected and counted after the 60 min pairing session. For each mating pair, the total number of eggs was divided by the number of separate *spawning* behaviors occurring in a 30 min period to determine an average number of eggs released by a single *spawning*; this average value was relatively reliable (low variance) with only a few outliers (Fig. 1E; 14.9 ± 4.3).

*Spawning* consists of gamete release by a male and a female. While the released eggs can be visualized, release of sperm could not be visualized even under high-speed camera. We reasoned that the percentage of fertilized eggs in released eggs correlate with coordinated *spawning*, which needs to occur in a short time window for successful fertilization.

We plotted the number of released eggs against that of fertilized eggs from multiple pairing sessions (Fig. 1F). The plot showed a strong, positive correlation (r = 0.81), however, the fertilization success rate was fairly constant (r = 0.075) when plotted against the total number of released eggs (Fig. 1G). These data suggest that the coordinated timing of *spawning* inferred from the fertilization rate were consistently observed and reliable.

Next, we examined how steps preceding *spawning* affected fertilization. Slow-motion videos showed that *quivering* constantly preceded *hooking*, which was regularly followed by *spawning*. In statistical analysis, the number of separate *hooking* bouts observed in 30 min correlated reliably with that of *quivering* (Fig. 2A, r = 0.85). *Hooking* showed moderately strong correlation with *spawning* (Fig. 2B, r = 0.68). These data suggest that the sequential steps of *quivering*, *hooking*, and *spawning* are tightly linked, and follow through once initiated.

To examine the significance of *hooking*, we contrived a method to specifically inhibit it. We predicted the female dorsal fin as a key for transition from *hooking* to *squeezing* because it seemed to constitute an integral element of the behavior (Fig. 1). Therefore, we surgically removed the dorsal fin from n=10 female zebrafish, of which we observed following their recovery period (Movie 3, Fig. 2C,D). We observed that without the female dorsal fin intact, the male trunk slipped rostrally and appeared to miss the opportunity to engage in *squeezing*. In response to such maladapted *hooking* and absent *squeezing*, the female terminated the behavioral mating sequence by evading the male without *spawning*.

We compared the number of *spawning* episodes exhibited by the same mating pairs, both before and after the female’s dorsal fin removal. The results showed a dramatical decrease after the fin removal (Fig. 2E; 25.1 ± 5.1 vs 3.5 ± 1.9; n=10), suggesting that *hooking* is critical for *spawning*. When the total released egg number was compared, it was also strongly decreased (Fig. 2F; 267 ± 49 vs 67 ± 31). Interestingly, among the small number of released eggs obtained after surgery, the fertilization rate was also reduced (Fig. 2G, 68 ± 5.7% vs 35 ± 11%), which suggested that the successful *hooking* is important for coordinating the timing of *spawning* between the male and female, as well as for adjusting the relative location of their gamete release.

We examined whether the dorsal fin removal also affects upstream steps in the behavioral mating sequence. The number of *quivering* bouts did not change between before, and after, the fin removal (Fig. 2H; 12.2 ± 3.9 vs 21.6 ± 7.8), suggesting that the failed *hooking* did not lead to compensatory increase of *quivering*. *Quivering* after surgery did lead to *freezing*, and subsequently to the incomplete *hooking* endeavor. The percentage of pairs proceeding from *quivering* to *hooking* (note *hooking* in these pairs ended uncompleted) did not change after the fin removal (Fig. 2I).

We next examined how *quivering* affects its downstream steps. We recently established a KO zebrafish line of the nicotinic acetylcholine receptor (AChR) ε subunit^14^. While the locomotion of εKOs were normal in many respects^14^, we found that the *quivering* of the KO male was compromised and used it as a tool to specifically impair *quivering* (Fig. 3A, Movie 4).

We analyzed the head angles during *quivering* using a high-speed camera (Fig. 3A). In WT pairs, the head angles plotted against time showed clear and regular peaks in accordance with previous studies^11^ (Fig. 3B). εKOs in contrast showed much reduced change in head angles. In addition, the pattern of the head turn was variable among individual males and many of them seemed uncoordinated because timing, direction, and degree of head angle change was unpredictable, unlike that of a WT zebrafish.

When the head angles shown in Fig. 3B were averaged (Fig. 3C), a clear oscillating pattern was evident in the WT male. In contrast, peaks were not recognizable in the εKO male, suggesting inconsistent phases. Because the regular oscillation could not be determined in many εKOs, the angle amplitude was determined automatically (see methods) and compared. The amplitude was strongly suppressed in εKOs compared to WTs (Fig. 3D; 48.9 ± 4.0 vs 24.0 ± 2.2 degrees).

Presumably due to the compromised *quivering*, pairs of the εKO male and the WT female did not lay eggs, even after 3 h of pairing (Fig. 3E). In these pairs, female did not show *freezing* response, and males did not display *hooking* behavior. We checked the fertility of εKO males by *in vitro* fertilization, using sperm from testis of a sacrificed εKO male and eggs from a WT female (Supplemental Fig. 2). Fertilization of eggs from this cross verified the quality of sperm in εKO males. In contrast, WT male and εKO female pairs showed typical mating behaviors and laid eggs, suggesting that the *freezing* performed by the εKO female was strong enough to cause following steps (Movie 5). Therefore, the εKO mutation appears to interrupt successful mating events by inhibiting the *quivering* courtship behavior in εKO males, yet not by inhibiting *freezing* or *spawning* behaviors by females.

## Discussion

**Figure 4:**
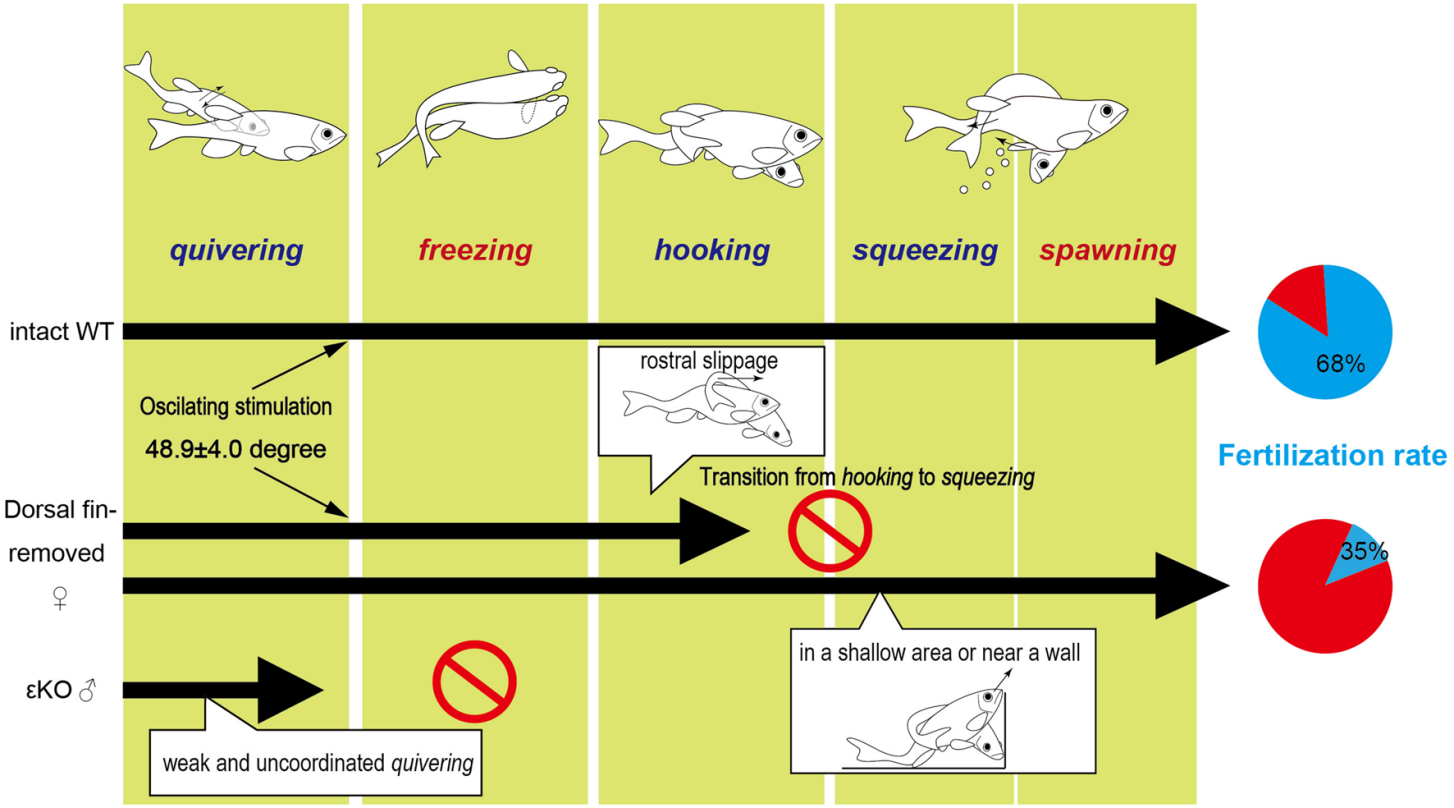
Summary. Mating of a typical zebrafish pair (intact, WT; Fig. 1) are compared with a pair that includes a female with the dorsal fin removal (without dorsal fin; Fig. 2) and a pair that includes the εKO male (εKO male; Fig. 3).

In the present study, based on high-speed motion analysis, we defined three previously uncharacterized steps of mating between the *quivering* and *spawning* behaviors: *freezing*, *hooking* and *squeezing* (Fig. 1A). For an efficient fertilization, it is important to synchronize the timing of *spawning*. We evaluated the synchronized *spawning* inferred from the fertilization rate, and examined the contribution of key steps using surgical intervention and genetic mutants (Fig. 4).

In response to *quivering* stimulations from WT males, WT female fish displayed S-shaped bending (*freezing*). WT females did not show *freezing* behavior to εKO males with insufficient *quivering* (Fig. 3, Movie 4). *Freezing* behavior may facilitate grasping of the female. Thus *freezing* as a receptive behavior may be equivalent to lordosis in female mice or rats^15,16^.

Behaviors similar to *freezing* have been reported in some teleosts. Female killifish (*Fundulus heteroclitus*) show S-shaped bending during *spawning* behavior, described as early as in 1907^17^. Later works suggested the S-shaped bending of killifish can be induced by injecting hormones understood to trigger reproductive behaviors, arginine vasopressin or oxytocin^18^, in the absence of *quivering*. S-shaped bending is also observed in the *spawning* behavior of medaka (*Oryzias latipes*), and the behavior in medaka also occurs in response to oxytocin (Atonin)^19^. Therefore, *freezing* behavior in zebrafish might be controlled by the same suite of reproductive hormones.

It is noteworthy, however, that in killifish and medaka, not only females but also males show S-shape bending during *spawning*, which is also induced by arginine vasopressin or oxytocin^18,19^. In contrast, male zebrafish never showed *freezing* or S-shaped bending during mating behavior. Moreover, the timing of S-shape bending in the mating process is different between medaka and zebrafish. Therefore, the significance and the control mechanism of the S-shaped bending are likely different between teleost species and there is more understanding to be uncovered regarding the courtship or mating behaviors of these traditionally medicinal and genetic model organisms.

Following *quivering*, the male fish transitioned to *hooking*. Removal of the female dorsal fin inhibited *hooking* (Fig. 2). However failed *hooking* occasionally led to *spawning*. These females spawned eggs in a shallow area in the tank (Fig. 4). Proximity to the floor may allow male fish to hold females without *hooking*.

Even when *spawning* occurred, fertilization rate in released eggs was strongly reduced after surgery (Fig. 2G). This is in sharp contrast to mating between WT pairs (Fig. 1F, G), in which the fertilization rate was independent from the clutch size. The release of eggs and sperm must be coordinated with regard to both timing and proximity. Our finding stresses the importance of *hooking* for triggering *spawning* behaviors.

A previous study suggested that surgically removing the pair of male pectoral fins, which are important for the grasping of the female, results in strikingly decreased fertilization rate^12^. In combination with the current study, males’ pectoral fins and females’ dorsal fins both seem important for coordinating fertilization. Any disturbance to the sequence of *hooking* and *squeezing* results in an erroneous coordination of *spawning* between the pair.

We analyzed *quivering* quantitatively, which has not been done previously. During *quivering*, the WT male directly stimulated the body of the female by repeatedly turning his head 48.9 ± 4.0 degrees. In addition to zebrafish, *quivering* behavior has been reported in other teleosts including medaka^20^, Mexican cavefish^21^, cichlid fish^22^, or salmoninae^23^. Medaka and Mexican cave fish males display *quivering* immediately preceding *spawning*. Thus, *quivering* in these species may be involved in inducing egg release by stimulating females in the same fashion as in zebrafish. In contrast, *quivering* in cichlid (*A. burtoni*) plays a different role^22^. A male cichlid fish escorts a female to his territory by displaying *quivering* in front of the female. After entering the* spawning* site, the pair encircle each other several times and lay eggs. Therefore, rather than inducing *spawning, quivering* in cichlids may function to attract females. All in all, *quivering* have varied functions across species. In the present study, using a mutant with impaired *quivering*, we showed that a male’s *quivering* behavior is necessary for the subsequent *freezing* behavior of females in zebrafish.

The εKO mutant does not express muscle-type nicotinic acetylcholine receptor (AChR) in fast muscles^14^. Although neuromuscular junctions in fast muscles are synaptically silenced, the εKO fish compensate their locomotion defects by rewiring motor neurons and converting slow muscles to fast muscles. Regardless of this neurological compensation, the εKO male fish showed defective *quivering* behavior. We speculate that *quivering* in the εKO males was weak and uncoordinated because of their smaller number of functional muscles compared to the WTs^12^. Indeed, while spontaneous swimming speed of εKOs is comparable to WTs^14^, the initial phase of escape response is weaker as measured by the amplitude of the head movement. Thus, the rapid succession of contractions during *quivering* may require increased muscle power, coordination, or energy compared to standard swimming. For the same reason, εKO male may not be able to perform *squeezing* either. However, it is difficult to examine this possibility because εKO male may not even attempt *squeezing,* which follows *quivering* in normal mating.

Conversely, εKO females paired with WT males showed typically successful *spawning* behavior in spite of the smaller number of functional muscles in εKO females^14^. This result suggests that female zebrafish need fewer functional muscle cells than males to complete *squeezing*, presumably due to the passive nature of the egg release by females. In medaka, females spawn simply by the *quivering* stimulation without *squeezing*^20^. When medaka pairs were disturbed during *quivering* by tapping on the tank, *quivering* stimulation longer than 4 sec was sufficient for female to start releasing eggs. Moreover, after* spawning* was initiated, females did not stop releasing eggs even when separated from males. One possible mechanism underlying this difference between zebrafish and medaka is the ovarian contraction, reported in several teleosts including medaka^21^, guppy^22^, killifish^23^ among others. The ovary contraction, induced by applied acetylcholine^24^, plays an important role in releasing eggs. In medaka, acetylcholine induces ovary contraction even after removal of the abdominal wall^25^, which suggests a direct stimulation of the ovarian smooth muscle. The ovary contraction has not been reported in zebrafish. Likewise, Mexican cavefish display *wrap around* behavior and *squeezing*-like behavior^26^, in which ovary contraction has not been reported. It is therefore a reasonable hypothesis that teleost species require one of two mechanisms to release eggs: *squeezing* behavior or ovary contraction. A curious next step in this investigation will involve examining whether the ovary of zebrafish or Mexican cavefish contract in response to acetylcholine. In addition, morphological analysis on smooth muscles in the ovary wall of various teleost species, including zebrafish and Mexican cavefish, will also provide important information to further understand the mechanism of egg release during *spawning* behavior.

## Supplementary Information

Supplementary Video 1.

Supplementary Video 2.

Supplementary Video 3.

Supplementary Video 4.

Supplementary Video 5.

Supplementary Information 1.
