# Supplementary material for: High-speed camera recordings uncover previously unidentified elements of zebrafish mating behaviors integral to successful fertilization
Source: Sci Rep. 2021 Oct 12;11:20228. doi: 10.1038/s41598-021-99638-6 (PMC8511115; doi:10.1038/s41598-021-99638-6)
Supplement: Supplementary file 6 — Supplementary Information 1. [file 41598_2021_99638_MOESM6_ESM.pdf]

Supplemental information

High-speed camera recordings uncover previously unidentified elements of zebrafish mating behaviors integral to successful fertilization

Buntaro Zempo[1,2], Natsuko Tanaka[1], Eriko Daikoku[1], and Fumihito Ono[1].

[1]Department of Physiology, Osaka Medical and Pharmaceutical University, Takatsuki 569-8686, Japan.

[2]Division of Integrative Physiology, Department of Physiology, Jichi Medical University, Shimotsuke 329-0498, Japan.

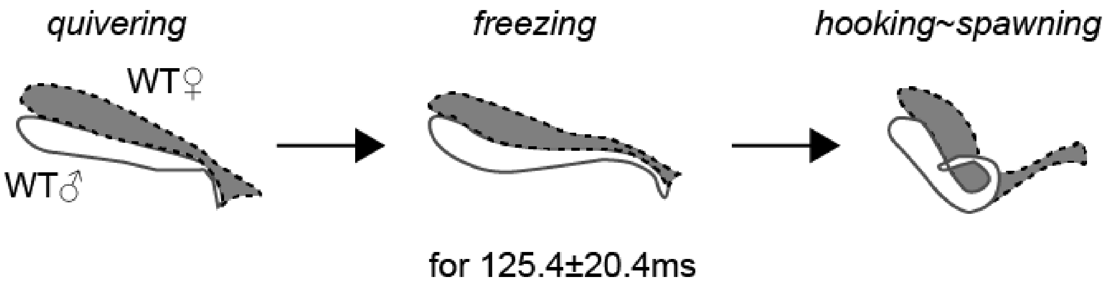

Supplemental Fig.1 *freezing* behavior

Illustrations show the process of mating ranging from *quivering* to *spawning* in a WT pair. Broken lines represent the outline of a female (filled grey), and solid lines represent the outline of a male (filled white). In response to *quivering*, the female displayed *freezing* behavior for 125.4 ± 20.4 ms (n=5) before the male proceeds to *hooking*. During this period, females did not display active movement except for passive displacement.

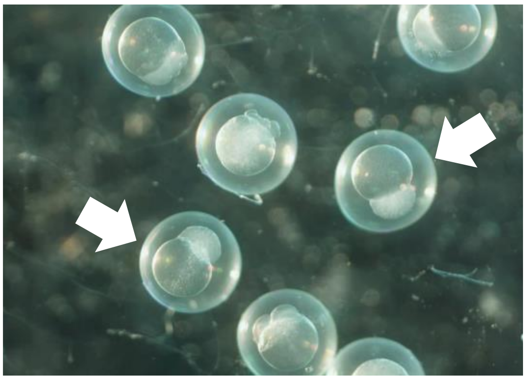

Supplemental Fig.2 *In vitro* fertilization evaluating the quality of sperms in an εKO male.

A photograph showing the eggs 2-3 hours after *in vitro* fertilization, using eggs from a WT female and sperms from an εKO male. 4 out of 12 eggs were fertilized and developed normally. Arrows indicate fertilized eggs.

Supplementary method

- *in vitro* fertilization

An εKO male was deeply anesthetized with tricaine before decapitation. Testis was isolated and homogenized in 10% N,N-Dimethylformamite (Nacalai tesque, Kyoto, Japan)-standard fetal bovine serum (Cytiva, Tokyo, Japan) solution on ice. A mature female WT fish was anesthetized with tricaine, and unfertilized eggs were squeezed out by gently pressing the abdomen. The sperm solution was subsequently applied to the eggs. Egg water was added to stimulate the motility of sperms.
